# Ethyl 5-methyl-3-[11-(pyridin-2-yl)-6,11-di­hydro-6,11-ep­oxy­dibenzo[*b*,*e*]oxepin-6-yl]isoxazole-4-carboxylate: a bicyclic acetal from the rearrangement of an anthracenyl isoxazole

**DOI:** 10.1107/S2056989020014358

**Published:** 2020-11-06

**Authors:** Matthew J. Weaver, Michael J. Campbell, Chun Li, Nicholas R. Natale

**Affiliations:** a Department of Biomedical & Pharmaceutical Sciences, University of Montana, Missoula, MT 59812, USA; b Department of Chemistry, Ithaca College, 953 Danby Road, Ithaca, NY 14850, USA

**Keywords:** crystal structure, ep­oxy­dibenzo[*b,e*]oxepin, isoxazole, anthracenyl isoxazole

## Abstract

The title compound, C_26_H_20_N_2_O_5_, is a rearrangement product of an *o*-pyridinyl anthracenyl isoxazole ester. It features a bicyclic acetal structure, which has two extended almost co-planar ring systems, which subtend a fold angle of 102.17 (5)°. In the crystal, the mol­ecules are closely knitted together through C—H⋯N and C—H⋯O hydrogen bonds and form chains of alternating enanti­omers propagating along the *c-*axis direction.

## Abstract 2

The title compound, a rearrangement product of an *o*-pyridinyl anthracenyl isoxazole ester, features a bicyclic acetal structure, which has two extended almost co-planar ring systems, which subtend a fold angle of 102.17 (5)°. In the crystal, the mol­ecules are closely knitted together through C—H⋯N and C—H⋯O hydrogen bonds and form chains of alternating enanti­omers propagating along the *c-*axis direction.

## Chemical context   

**Figure 1:**
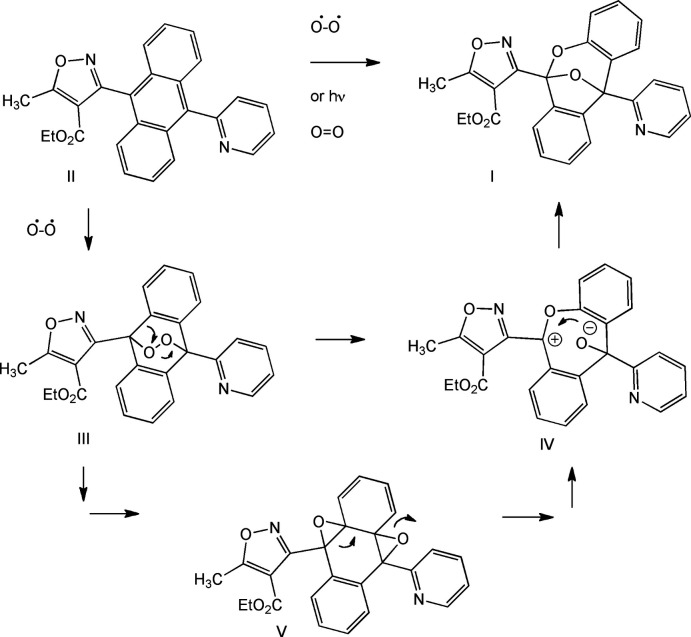
Rearrangement of *o*-pyridyl ester **II**. The title compound **I** was observed on slow evaporation during recrystallization at room temperature.

We have reported on 3-aryl isoxazole amides (AIMs) with anti­tumor activity (Han *et al.*, 2009 ▸; Weaver *et al.*, 2015 ▸) and recently described 10-substituted anthracenes with N-heterocyclic substituents in this series, which possessed robust anti­tumor activity against both breast and brain tumor cell lines (Weaver *et al.*, 2020 ▸). In the course of that study, we attempted to obtain crystals of the 10-*o*-pyridyl example **II** by slow evaporation (see Fig. 1 ▸). After numerous attempts, suitable crystals were obtained but were found to have undergone oxygen addition and rearrangement to the title compound, C_26_H_20_N_2_O_5_, **I**. This is unprecedented in this series of compounds.
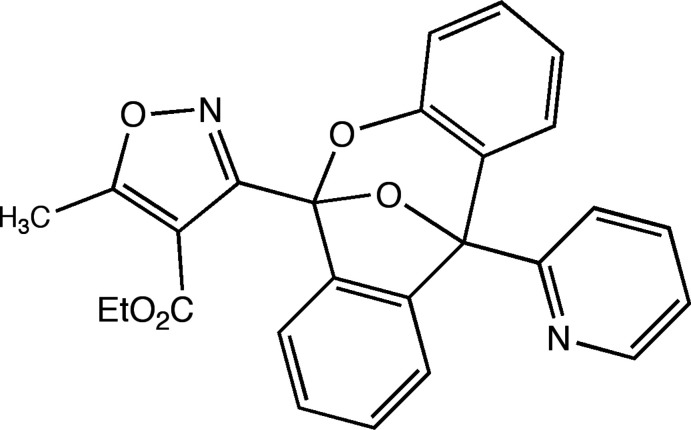


In the case of the *o*-pyridyl ester, slow evaporation from solution was observed to produce a bicyclic acetal (BA). This requires the formation of a di­oxy­gen adduct commonly found in the anthracene literature (Klaper *et al.*, 2016 ▸), as shown in Fig. 1 ▸. This di­oxy­gen adduct **III** is most often observed as a [4 + 2] cyclo­adduct with singlet oxygen (Lauer *et al.*, 2011 ▸), and in some cases where a donor–acceptor pair sensitizes the formation of singlet oxygen. It should be noted, however, that the endo peroxide can be formed from the ground-state diradical oxygen in a one-electron process.

The bicyclic acetal (BA) **I** can be formed directly *via* a Criegee-like rearrangement through inter­mediate **IV**, or alternatively stepwise *via* the inter­mediacy of one electron reorganization to an inter­mediate diepoxide **V** (Filatov *et al.*, 2017 ▸). Of the ten previous crystal structures of anthryl isoxazoles published by our group (Mosher *et al.*, 1996 ▸; Han *et al.*, 2002 ▸, 2003 ▸; Li *et al.*, 2006 ▸, 2008 ▸; Li *et al.*, 2013 ▸; Duncan *et al.*, 2014 ▸; Weaver *et al.*, 2015 ▸), and the three N-heterocyclic structures solved and disclosed (Weaver *et al.*, 2020 ▸), this is the first example we have observed of this rearrangement. Given the observation of this rearrangement it is advisable that the *o*-pyridyl AIM (**II**) be stored under an argon atmosphere at low temperature (233 K or below).

Conditions within tumors are notoriously anoxic. As an example, the transition to the Warberg phenotype (Vander Heiden *et al.*, 2009 ▸) is heavily influenced by the transcription factor hypoxia inducing factor (HIF). Therefore, the physiological relevance and therapeutic practicality of this process appears questionable, particularly considering that the *endo* peroxide (**III**) or the diepoxide (**V**) would not be expected to exert significant selectivity. Therefore, the probability of a useful therapeutic index would appear low. However, the prospects for exploiting this tactic will be considered, even if they constitute only negative controls, in our ongoing studies of anti­tumor theranostics, and will be reported in due course.

## Structural commentary   

**Figure 2:**
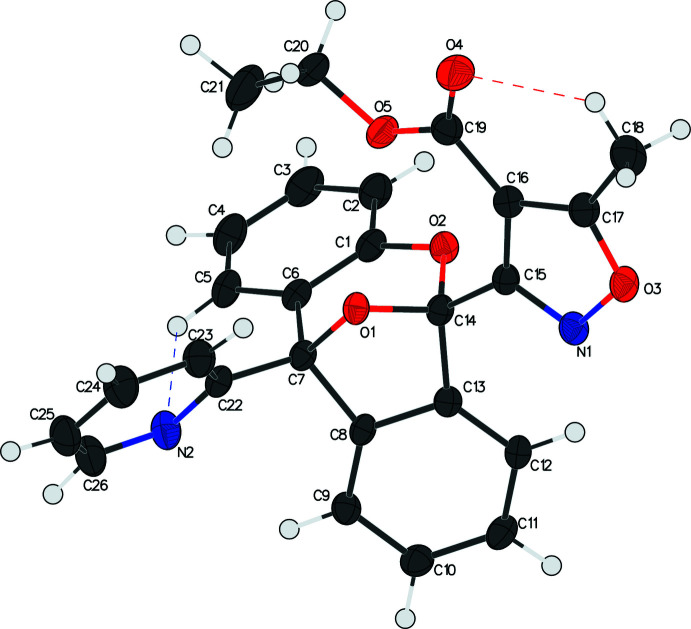
The mol­ecular structure of **I** showing the atom-labeling scheme. Displacement ellipsoids are drawn at the 50% probability level. Dashed lines indicate intra­molecular hydrogen bonds.

The title compound crystallizes as a racemate in the monoclinic space group, *P*2_1_/c, with one independent mol­ecule in the asymmetric unit (Fig. 2 ▸). In the arbitrarily chosen asymmetric mol­ecule, atoms C7 and C14 both have *R* configurations. The insertion of two oxygen atoms in the central ring of anthracene forms a bicyclic system with one oxygen atom (O1) in the middle shared by both dioxane and furan rings. The remainder of the dioxane and furan ring atoms are co-planar with the C1–C6 and C8–C13 benzene rings on either side, respectively. The pyridine group is attached at the *ortho* position to one of the shared carbon atoms on the bicyclic system, while the isoxazole ester is attached to the other shared carbon atom. The overall effect of the bonding gives the whole mol­ecule a dragon-like appearance.

The planarity of each wing is indicated by the r.m.s.d. of 0.028 Å for both planes formed by C1–C7/C14/O2 and C7–C14. These two wings are flapping downwards with a fold angle between them of 102.17 (5)°. The pyridine group is the head of the dragon with the nitro­gen atom being *exo* to the oxygen atom (O1) in the backbone. A potential hydrogen bond between C23—H23 and O1 may contribute to the small torsion angle of 2.2 (3)° for O1—C7—C22—C23. Both the nitro­gen and oxygen atoms in the isoxazole ring are *exo* to the oxygen atoms (O1 and O2) in the dioxane ring, resulting in the ethyl ester tail swinging to the dioxane side and coming to rest between the two oxygen atoms. There is a σ–π inter­action between the tip of the tail (methyl group) and the benzene ring, which is also reflected by the upfield shift of CH_3_ protons in the NMR spectrum.

## Supra­molecular features   

**Figure 3:**
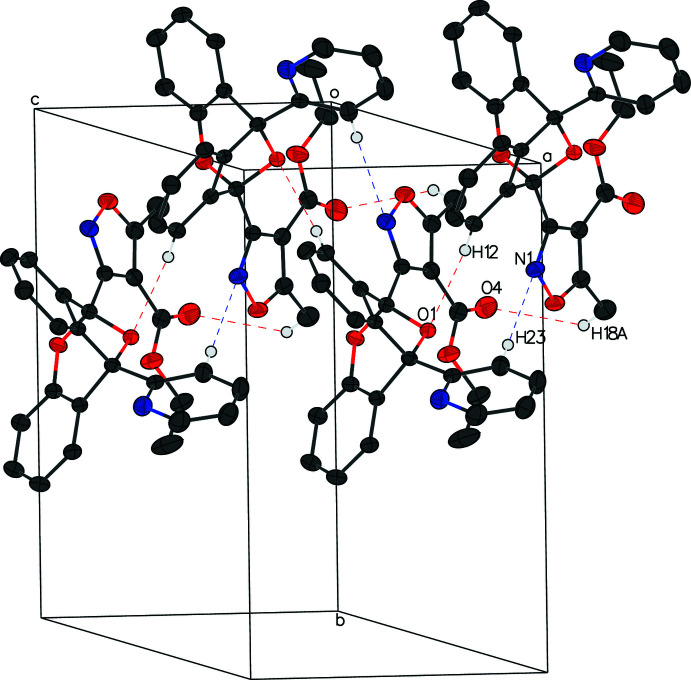
The packing of **I**. A closely knitted chain of alternating enanti­omers is formed through several inter­molecular hydrogen bonds. For clarity, H atoms not participating in inter­molecular bonds are omitted. Atoms participating in inter­molecular hydrogen bonds are labeled once.

In the crystal, chains of alternating enanti­omers are formed running along the *c*-axis direction through the inter­molecular hydrogen bonds C23—H23⋯N1^i^, C12—H12⋯O1^ii^ and C18—H18*B*⋯O4^ii^ (Table 1 ▸, Fig. 3 ▸). This chain is highly knitted, which may contribute to the formation of needle-shaped crystals.

## Hirshfeld surface analysis   

**Figure 4:**
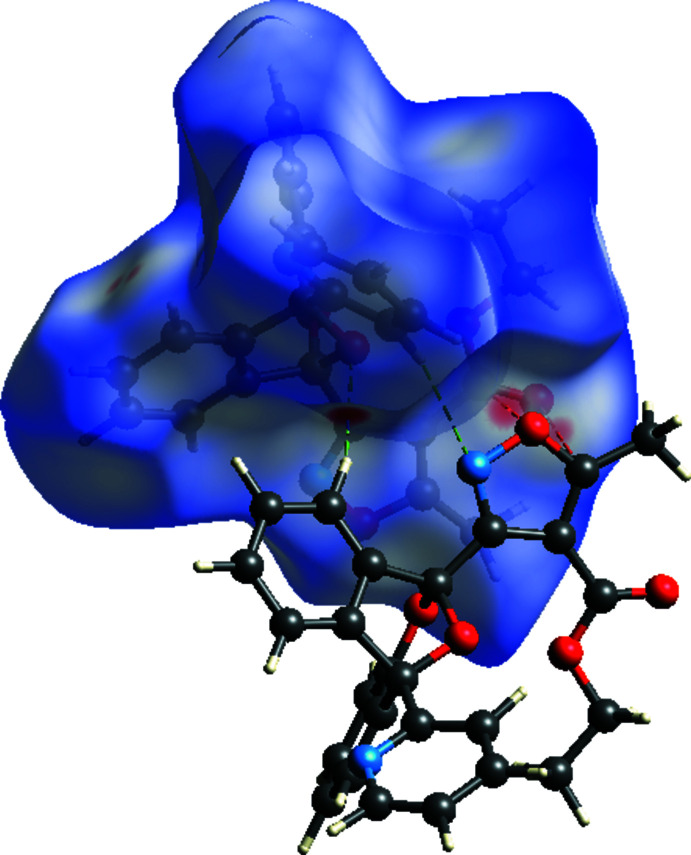
Hirshfeld surface of **I** mapped over *d*_norm_. Short contacts between carbonyl C19=O3 and isoxazole O3—C17 are shown in dashed red lines. Inter­molecular hydrogen bonds O1⋯H12 and N1⋯H23 are shown as dashed green lines.

**Figure 5:**
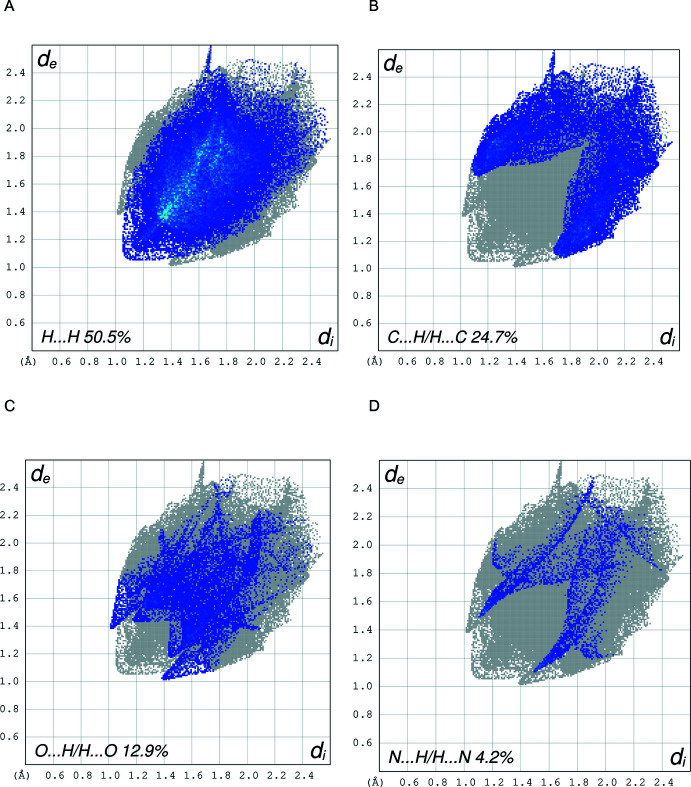
The two-dimensional fingerprint plots for **I** delineated into (*a*) H⋯H/H⋯H contacts, (*b*) C⋯H/H⋯C contacts, (*c*) O⋯H/H⋯O contacts, and (*d*) N⋯H/H⋯N contacts.

**Figure 6:**
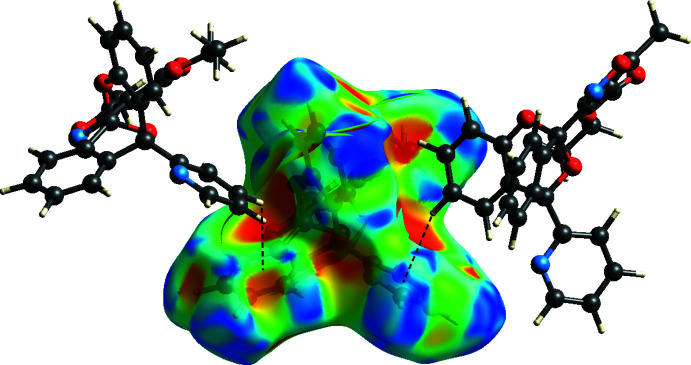
Hirshfeld surface of **I** mapped over the shape-index property. C—H⋯π inter­actions (C24—H24 to aromatic ring C1–C6 and C4—H4 to aromatic ring N22/C22–C26) are shown as dashed lines.

The inter­molecular inter­actions were qu­anti­fied using Hirshfeld surface analysis (Spackman & Jayatilaka, 2009 ▸) and the associated two-dimensional fingerprint plots (McKinnon *et al.*, 2007 ▸). The calculations and visualization were performed using *CrystalExplorer17* (Turner *et al.*, 2017 ▸). The Hirshfeld surface of the title compound is mapped over *d*_norm_ in a fixed color scale of −0.1374 (red) to +1.3125 (blue) arbitrary units (Fig. 4 ▸), where the red spots indicate the inter­molecular contacts shorter than the van der Waals separations. The delineated two-dimensional fingerprint plots are shown in Fig. 5 ▸, and demonstrate that the main contribution to the overall Hirshfeld surface area arises from H⋯H contacts (50.5%, Fig. 5 ▸ *a*). The C⋯H/H⋯C contacts (24.7%, Fig. 5 ▸ *b*), which indicate C—H⋯π inter­actions, are identifiable from the Hirshfeld surface mapped over the shape-index property (Fig. 6 ▸). Conventional hydrogen-bonding inter­actions, H⋯O/O⋯H and N⋯H/H⋯N, only comprise 12.9% and 4.2% of the inter­molecular inter­actions, respectively (Fig. 5 ▸ *b* and 5*c*).

## Database survey   

A search for the 6,11-di­hydro-6,11-ep­oxy­dibenzo[*b,e*]oxepin fragment in the Cambridge Structural Database (CSD version 5.40, August 2019 update; Groom *et al.*, 2016 ▸) resulted in five hits, namely refcodes LIPZEP (Walker *et al.*, 1999 ▸), NEJLOG (Filatov *et al.*, 2017 ▸), VAZDEI, VAZDIM (Ando *et al.*, 2017 ▸), and WOPGAM (Ando *et al.*, 2019 ▸). These five structures, despite their different substitution groups and positions, all exhibit a similar a structural configuration, that with shared oxygen atom pointing up, and the remainder of the five- and seven-membered rings on the bicyclic system are co-planar to their respective benzene rings.

## Synthesis and crystallization   

The title compound was synthesized from the *o*-pyridyl-anthracenyl isoxazole ester (**II**) (Weaver *et al.*, 2020 ▸). Colorless needles were obtained by slow evaporation in the presence of atmospheric oxygen over a period of several months. ^1^H NMR (CDCl_3_) δppm 8.895 (*d*, 1H, *J* = 5Hz); 8.13 (*dd*, 1H, *J* = 6 Hz); 7.8 (*d*, 2H, *J* = 4 Hz); 7.57 (*dt*, 1H); 7.34 (*m*, 4H); 7.16 (*m*, 1H); 6.85 (*d*, 1H, 8 Hz); 6.79 (*t*, 1H, *J* = 8 Hz); 3.975 (*q*, 1H, *J* = 7 Hz); 3.845 (*q*, 1H, *J* = 7 Hz); 2.79 (*s*, 3H); 0.63 (*t*, 3H, *J* = 7 Hz). ^13^C NMR (CDCl_3_) δppm 176.69, 161.73 158.05, 156.86, 150.24, 148.55, 146.15, 135.98, 136.88, 129.35, 128.44, 128.17, 123.27, 123.09, 122.16, 121.46, 121.35, 120.46, 117.01, 60.91, 13.14. 13.08. The proton–proton correlation is provided in the supporting information. Positive electrospray ionization (ESI) mass spectrometry, calc. for [C_26_H_20_N_2_O_3_+H]^+^ 441.44, observed *m*/*z* 441.2 ([*M *+ H]^+^, 100% rel. intensity).

## Refinement   

Crystal data, data collection and structure refinement details are summarized in Table 2 ▸. All hydrogen atoms were found in difference-Fourier maps and their positions were freely refined with the constraint *U*_iso_(H) = 1.2 or 1.5*U*_eq_(parent). Seven reflections were omitted because of poor agreement between the observed and calculated intensities.

## Supplementary Material

Crystal structure: contains datablock(s) I. DOI: 10.1107/S2056989020014358/hb7949sup1.cif

Structure factors: contains datablock(s) I. DOI: 10.1107/S2056989020014358/hb7949Isup2.hkl

Proton-Proton COSY of the title compound. DOI: 10.1107/S2056989020014358/hb7949sup3.pdf

Supporting information file. DOI: 10.1107/S2056989020014358/hb7949Isup4.cml

CCDC reference: 2041149

Additional supporting information:  crystallographic information; 3D view; checkCIF report

## Figures and Tables

**Table 1:** Hydrogen-bond geometry (Å, °)

*D*—H⋯*A*	*D*—H	H⋯*A*	*D*⋯*A*	*D*—H⋯*A*
C5—H5⋯N2	0.96 (2)	2.42 (2)	3.085 (3)	125.8 (18)
C23—H23⋯N1^i^	1.00 (3)	2.70 (3)	3.669 (3)	163 (2)
C12—H12⋯O1^ii^	0.94 (3)	2.56 (3)	3.469 (2)	162 (2)
C18—H18*A*⋯O4^ii^	0.94 (4)	2.71 (4)	3.297 (3)	121 (3)
C18—H18*B*⋯O4	0.99 (4)	2.38 (4)	3.040 (3)	123 (3)

Symmetry codes: (i) 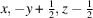; (ii) 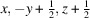.

**Table 2:** Experimental details

Crystal data	
Chemical formula	C_26_H_20_N_2_O_5_
*M*_r_	440.44
Crystal system, space group	Monoclinic, *P*2_1_/*c*
Temperature (K)	150
*a*, *b*, *c* (Å)	19.0586 (7), 13.9627 (5), 8.1459 (3)
β (°)	101.7800 (11)
*V* (Å^3^)	2122.05 (13)
*Z*	4
Radiation type	Synchrotron, λ = 0.7288 Å
μ (mm^−1^)	0.10
Crystal size (mm)	0.11 × 0.01 × 0.01

Data collection	
Diffractometer	Bruker PHOTON-II
Absorption correction	Multi-scan (*SADABS*; Krause *et al.*, 2015 ▸)
No. of measured, independent and observed [*I* > 2σ(*I*)] reflections	71254, 5275, 3947
*R*_int_	0.067
(sin θ/λ)_max_ (Å^−1^)	0.667

Refinement	
*R*[*F* ^2^ > 2σ(*F* ^2^)], *wR*(*F* ^2^), *S*	0.052, 0.145, 1.05
No. of reflections	5275
No. of parameters	378
H-atom treatment	All H-atom parameters refined
Δρ_max_, Δρ_min_ (e Å^−3^)	0.28, −0.27

Computer programs: *APEX3* and *SAINT* (Bruker, 2016 ▸), *SHELXS* (Sheldrick, 2008 ▸), *SHELXL* (Sheldrick, 2015 ▸), *OLEX2* (Dolomanov *et al.*, 2009 ▸), and *publCIF* (Westrip, 2010 ▸).

## supplementary crystallographic information

### Crystal data

**Table:**

C_26_H_20_N_2_O_5_	*F*(000) = 920
*M**_r_* = 440.44	*D*_x_ = 1.379 Mg m^−^^3^
Monoclinic, *P*2_1_/*c*	Synchrotron radiation, λ = 0.7288 Å
*a* = 19.0586 (7) Å	Cell parameters from 9997 reflections
*b* = 13.9627 (5) Å	θ = 2.7–29.0°
*c* = 8.1459 (3) Å	µ = 0.10 mm^−^^1^
β = 101.7800 (11)°	*T* = 150 K
*V* = 2122.05 (13) Å^3^	Needle, colourless
*Z* = 4	0.11 × 0.01 × 0.01 mm

### Data collection

**Table:**

Bruker PHOTON-II diffractometer	5275 independent reflections
Radiation source: synchrotron	3947 reflections with *I* > 2σ(*I*)
Si-<111> channnel cut crystal monochromator	*R*_int_ = 0.067
ω–phi scans	θ_max_ = 29.1°, θ_min_ = 1.1°
Absorption correction: multi-scan (SADABS; Krause *et al.*, 2015)	*h* = −25→25
	*k* = −18→18
71254 measured reflections	*l* = −10→10

### Refinement

**Table:**

Refinement on *F*^2^	Primary atom site location: structure-invariant direct methods
Least-squares matrix: full	Hydrogen site location: difference Fourier map
*R*[*F*^2^ > 2σ(*F*^2^)] = 0.052	All H-atom parameters refined
*wR*(*F*^2^) = 0.145	*w* = 1/[σ^2^(*F*_o_^2^) + (0.0476*P*)^2^ + 2.6861*P*] where *P* = (*F*_o_^2^ + 2*F*_c_^2^)/3
*S* = 1.05	(Δ/σ)_max_ < 0.001
5275 reflections	Δρ_max_ = 0.28 e Å^−^^3^
378 parameters	Δρ_min_ = −0.27 e Å^−^^3^
0 restraints	

### Special details

**Table:**

Geometry. All esds (except the esd in the dihedral angle between two l.s. planes) are estimated using the full covariance matrix. The cell esds are taken into account individually in the estimation of esds in distances, angles and torsion angles; correlations between esds in cell parameters are only used when they are defined by crystal symmetry. An approximate (isotropic) treatment of cell esds is used for estimating esds involving l.s. planes.

### Fractional atomic coordinates and isotropic or equivalent isotropic displacement parameters (Å^2^)

**Table:**

	*x*	*y*	*z*	*U*_iso_*/*U*_eq_	
O1	0.24461 (7)	0.07865 (10)	0.35674 (16)	0.0202 (3)	
O2	0.29900 (8)	0.07367 (10)	0.64004 (17)	0.0223 (3)	
O3	0.34820 (8)	0.35999 (10)	0.50102 (19)	0.0261 (3)	
O4	0.47276 (9)	0.15114 (12)	0.3200 (2)	0.0361 (4)	
O5	0.39223 (8)	0.05030 (11)	0.3916 (2)	0.0296 (4)	
N1	0.29207 (9)	0.29999 (12)	0.5258 (2)	0.0238 (4)	
N2	0.09144 (10)	−0.07696 (14)	0.2123 (2)	0.0292 (4)	
C1	0.27403 (12)	−0.01948 (14)	0.6403 (3)	0.0232 (4)	
C2	0.30636 (13)	−0.07614 (16)	0.7747 (3)	0.0287 (5)	
C3	0.28365 (14)	−0.17036 (16)	0.7839 (3)	0.0344 (5)	
C4	0.22965 (14)	−0.20738 (16)	0.6600 (3)	0.0326 (5)	
C5	0.19750 (13)	−0.14975 (15)	0.5254 (3)	0.0266 (5)	
C6	0.21931 (11)	−0.05535 (14)	0.5141 (2)	0.0211 (4)	
C7	0.18559 (10)	0.01676 (13)	0.3788 (2)	0.0193 (4)	
C8	0.14033 (11)	0.08444 (13)	0.4614 (2)	0.0193 (4)	
C9	0.06981 (11)	0.07996 (15)	0.4768 (3)	0.0228 (4)	
C10	0.04475 (12)	0.15003 (16)	0.5736 (3)	0.0263 (4)	
C11	0.09066 (12)	0.22102 (16)	0.6540 (3)	0.0272 (5)	
C12	0.16221 (11)	0.22441 (15)	0.6391 (2)	0.0226 (4)	
C13	0.18632 (11)	0.15600 (14)	0.5409 (2)	0.0197 (4)	
C14	0.26003 (11)	0.13279 (14)	0.5063 (2)	0.0201 (4)	
C15	0.31029 (11)	0.21370 (14)	0.4879 (2)	0.0202 (4)	
C16	0.37804 (11)	0.21339 (15)	0.4366 (2)	0.0227 (4)	
C17	0.39812 (12)	0.30763 (15)	0.4467 (3)	0.0249 (4)	
C18	0.46053 (15)	0.3608 (2)	0.4110 (4)	0.0355 (5)	
C19	0.41961 (11)	0.13631 (16)	0.3764 (3)	0.0254 (4)	
C20	0.42398 (14)	−0.02839 (17)	0.3144 (3)	0.0330 (5)	
C21	0.38260 (19)	−0.1163 (2)	0.3364 (5)	0.0555 (9)	
C22	0.14987 (11)	−0.02307 (14)	0.2102 (2)	0.0214 (4)	
C23	0.17449 (13)	−0.00322 (17)	0.0645 (3)	0.0285 (5)	
C24	0.13755 (14)	−0.04157 (19)	−0.0852 (3)	0.0344 (5)	
C25	0.07809 (14)	−0.09848 (18)	−0.0845 (3)	0.0347 (5)	
C26	0.05705 (14)	−0.11353 (18)	0.0655 (3)	0.0343 (5)	
H5	0.1576 (13)	−0.1717 (17)	0.441 (3)	0.023 (6)*	
H4	0.2093 (14)	−0.276 (2)	0.662 (3)	0.037 (7)*	
H24	0.1525 (15)	−0.028 (2)	−0.187 (4)	0.038 (7)*	
H3	0.3064 (15)	−0.214 (2)	0.884 (4)	0.042 (8)*	
H25	0.0550 (15)	−0.128 (2)	−0.186 (4)	0.042 (8)*	
H23	0.2149 (15)	0.043 (2)	0.067 (3)	0.037 (7)*	
H20A	0.4748 (15)	−0.0340 (19)	0.366 (3)	0.034 (7)*	
H9	0.0375 (14)	0.0326 (19)	0.423 (3)	0.029 (6)*	
H20B	0.4242 (18)	−0.013 (2)	0.194 (4)	0.060 (9)*	
H2	0.3400 (15)	−0.053 (2)	0.861 (4)	0.036 (7)*	
H10	−0.0068 (16)	0.148 (2)	0.583 (4)	0.045 (8)*	
H26	0.0124 (16)	−0.152 (2)	0.070 (3)	0.043 (8)*	
H11	0.0758 (14)	0.269 (2)	0.723 (3)	0.036 (7)*	
H12	0.1939 (14)	0.272 (2)	0.692 (3)	0.037 (7)*	
H18A	0.492 (2)	0.386 (3)	0.506 (5)	0.080 (12)*	
H21A	0.332 (2)	−0.108 (2)	0.279 (4)	0.062 (10)*	
H18B	0.495 (2)	0.317 (3)	0.373 (5)	0.079 (12)*	
H21B	0.403 (2)	−0.175 (3)	0.273 (5)	0.097 (14)*	
H18C	0.451 (2)	0.395 (3)	0.322 (6)	0.095 (15)*	
H21C	0.385 (2)	−0.129 (3)	0.471 (5)	0.080 (12)*	

### Atomic displacement parameters (Å^2^)

**Table:**

	*U*^11^	*U*^22^	*U*^33^	*U*^12^	*U*^13^	*U*^23^
O1	0.0246 (7)	0.0169 (6)	0.0200 (6)	−0.0032 (5)	0.0065 (5)	−0.0011 (5)
O2	0.0264 (7)	0.0168 (7)	0.0225 (7)	−0.0009 (6)	0.0024 (6)	0.0027 (5)
O3	0.0292 (8)	0.0186 (7)	0.0320 (8)	−0.0061 (6)	0.0096 (6)	−0.0011 (6)
O4	0.0313 (9)	0.0360 (9)	0.0452 (10)	−0.0020 (7)	0.0176 (8)	−0.0050 (8)
O5	0.0284 (8)	0.0203 (7)	0.0424 (9)	0.0007 (6)	0.0124 (7)	−0.0039 (6)
N1	0.0246 (9)	0.0199 (8)	0.0274 (9)	−0.0048 (7)	0.0065 (7)	0.0007 (7)
N2	0.0326 (10)	0.0281 (9)	0.0279 (9)	−0.0085 (8)	0.0084 (8)	−0.0047 (7)
C1	0.0294 (11)	0.0172 (9)	0.0246 (10)	0.0018 (8)	0.0094 (8)	0.0012 (7)
C2	0.0367 (12)	0.0226 (10)	0.0264 (11)	0.0027 (9)	0.0053 (9)	0.0019 (8)
C3	0.0497 (15)	0.0225 (11)	0.0317 (12)	0.0083 (10)	0.0097 (11)	0.0078 (9)
C4	0.0482 (14)	0.0171 (10)	0.0339 (12)	0.0009 (10)	0.0120 (10)	0.0030 (9)
C5	0.0367 (12)	0.0173 (10)	0.0276 (10)	−0.0015 (8)	0.0110 (9)	−0.0017 (8)
C6	0.0266 (10)	0.0168 (9)	0.0218 (9)	0.0017 (8)	0.0093 (8)	0.0006 (7)
C7	0.0226 (10)	0.0148 (9)	0.0220 (9)	−0.0017 (7)	0.0078 (7)	0.0004 (7)
C8	0.0254 (10)	0.0138 (8)	0.0196 (9)	−0.0005 (7)	0.0067 (7)	0.0007 (7)
C9	0.0244 (10)	0.0200 (10)	0.0246 (10)	−0.0038 (8)	0.0068 (8)	0.0001 (8)
C10	0.0250 (11)	0.0265 (11)	0.0293 (11)	0.0000 (8)	0.0104 (9)	−0.0008 (8)
C11	0.0319 (12)	0.0235 (10)	0.0289 (10)	0.0021 (9)	0.0122 (9)	−0.0045 (8)
C12	0.0273 (11)	0.0185 (9)	0.0219 (9)	−0.0018 (8)	0.0046 (8)	−0.0029 (7)
C13	0.0231 (10)	0.0174 (9)	0.0190 (9)	−0.0011 (7)	0.0049 (7)	0.0015 (7)
C14	0.0251 (10)	0.0167 (9)	0.0184 (9)	0.0003 (8)	0.0040 (7)	0.0007 (7)
C15	0.0238 (10)	0.0176 (9)	0.0188 (9)	−0.0009 (7)	0.0033 (7)	−0.0001 (7)
C16	0.0245 (10)	0.0224 (10)	0.0211 (9)	−0.0012 (8)	0.0049 (8)	0.0009 (8)
C17	0.0277 (11)	0.0232 (10)	0.0239 (10)	−0.0024 (8)	0.0057 (8)	0.0003 (8)
C18	0.0354 (13)	0.0327 (13)	0.0409 (14)	−0.0113 (11)	0.0136 (11)	−0.0006 (11)
C19	0.0249 (11)	0.0271 (11)	0.0242 (10)	−0.0006 (8)	0.0052 (8)	−0.0016 (8)
C20	0.0318 (13)	0.0261 (11)	0.0424 (13)	0.0051 (9)	0.0106 (10)	−0.0080 (10)
C21	0.055 (2)	0.0274 (13)	0.093 (3)	−0.0047 (13)	0.0343 (19)	−0.0172 (15)
C22	0.0266 (10)	0.0152 (9)	0.0230 (9)	0.0007 (7)	0.0060 (8)	−0.0010 (7)
C23	0.0332 (12)	0.0287 (11)	0.0244 (10)	−0.0029 (9)	0.0078 (9)	−0.0019 (8)
C24	0.0412 (14)	0.0411 (14)	0.0219 (10)	−0.0040 (11)	0.0089 (10)	−0.0041 (9)
C25	0.0369 (13)	0.0380 (13)	0.0270 (11)	−0.0046 (10)	0.0012 (10)	−0.0094 (10)
C26	0.0344 (13)	0.0339 (12)	0.0339 (12)	−0.0100 (10)	0.0052 (10)	−0.0083 (10)

### Geometric parameters (Å, º)

**Table:**

O1—C7	1.458 (2)	C10—C11	1.394 (3)
O1—C14	1.413 (2)	C10—H10	1.00 (3)
O2—C1	1.385 (2)	C11—C12	1.394 (3)
O2—C14	1.446 (2)	C11—H11	0.95 (3)
O3—N1	1.405 (2)	C12—C13	1.383 (3)
O3—C17	1.344 (3)	C12—H12	0.94 (3)
O4—C19	1.212 (3)	C13—C14	1.523 (3)
O5—C19	1.325 (3)	C14—C15	1.508 (3)
O5—C20	1.457 (3)	C15—C16	1.436 (3)
N1—C15	1.308 (3)	C16—C17	1.368 (3)
N2—C22	1.347 (3)	C16—C19	1.478 (3)
N2—C26	1.342 (3)	C17—C18	1.480 (3)
C1—C2	1.389 (3)	C18—H18A	0.94 (4)
C1—C6	1.399 (3)	C18—H18B	0.99 (4)
C2—C3	1.392 (3)	C18—H18C	0.86 (5)
C2—H2	0.91 (3)	C20—C21	1.489 (4)
C3—C4	1.386 (4)	C20—H20A	0.98 (3)
C3—H3	1.04 (3)	C20—H20B	1.00 (3)
C4—C5	1.397 (3)	C21—H21A	1.00 (4)
C4—H4	1.03 (3)	C21—H21B	1.08 (4)
C5—C6	1.391 (3)	C21—H21C	1.11 (4)
C5—H5	0.96 (2)	C22—C23	1.391 (3)
C6—C7	1.534 (3)	C23—C24	1.385 (3)
C7—C8	1.525 (3)	C23—H23	1.00 (3)
C7—C22	1.509 (3)	C24—C25	1.385 (4)
C8—C9	1.377 (3)	C24—H24	0.95 (3)
C8—C13	1.398 (3)	C25—C26	1.378 (3)
C9—C10	1.400 (3)	C25—H25	0.94 (3)
C9—H9	0.95 (3)	C26—H26	1.02 (3)
			
C14—O1—C7	103.92 (14)	O1—C14—C15	109.84 (15)
C1—O2—C14	114.43 (15)	O2—C14—C13	109.49 (15)
C17—O3—N1	109.49 (15)	O2—C14—C15	105.29 (15)
C19—O5—C20	115.89 (17)	C15—C14—C13	119.18 (16)
C15—N1—O3	105.57 (16)	N1—C15—C14	117.49 (17)
C26—N2—C22	117.14 (19)	N1—C15—C16	111.68 (18)
O2—C1—C2	116.04 (19)	C16—C15—C14	130.82 (18)
O2—C1—C6	122.66 (18)	C15—C16—C19	132.33 (19)
C2—C1—C6	121.30 (19)	C17—C16—C15	103.68 (18)
C1—C2—C3	119.2 (2)	C17—C16—C19	123.92 (19)
C1—C2—H2	122.2 (18)	O3—C17—C16	109.57 (18)
C3—C2—H2	118.5 (17)	O3—C17—C18	116.29 (19)
C2—C3—H3	120.6 (16)	C16—C17—C18	134.1 (2)
C4—C3—C2	120.4 (2)	C17—C18—H18A	115 (2)
C4—C3—H3	119.0 (16)	C17—C18—H18B	112 (2)
C3—C4—C5	119.9 (2)	C17—C18—H18C	114 (3)
C3—C4—H4	124.1 (15)	H18A—C18—H18B	98 (3)
C5—C4—H4	115.9 (15)	H18A—C18—H18C	118 (4)
C4—C5—H5	122.1 (14)	H18B—C18—H18C	97 (3)
C6—C5—C4	120.6 (2)	O4—C19—O5	124.4 (2)
C6—C5—H5	117.2 (14)	O4—C19—C16	123.2 (2)
C1—C6—C7	115.67 (17)	O5—C19—C16	112.39 (18)
C5—C6—C1	118.60 (19)	O5—C20—C21	107.0 (2)
C5—C6—C7	125.64 (19)	O5—C20—H20A	109.8 (16)
O1—C7—C6	104.89 (15)	O5—C20—H20B	110.4 (19)
O1—C7—C8	101.96 (14)	C21—C20—H20A	112.7 (16)
O1—C7—C22	108.79 (15)	C21—C20—H20B	113.4 (19)
C8—C7—C6	106.35 (15)	H20A—C20—H20B	104 (2)
C22—C7—C6	117.20 (16)	C20—C21—H21A	110 (2)
C22—C7—C8	116.05 (17)	C20—C21—H21B	109 (2)
C9—C8—C7	131.50 (18)	C20—C21—H21C	110 (2)
C9—C8—C13	121.45 (18)	H21A—C21—H21B	106 (3)
C13—C8—C7	106.83 (17)	H21A—C21—H21C	109 (3)
C8—C9—C10	118.11 (19)	H21B—C21—H21C	114 (3)
C8—C9—H9	123.0 (15)	N2—C22—C7	114.51 (17)
C10—C9—H9	118.8 (15)	N2—C22—C23	123.09 (19)
C9—C10—H10	118.4 (17)	C23—C22—C7	122.37 (18)
C11—C10—C9	120.5 (2)	C22—C23—H23	120.0 (16)
C11—C10—H10	121.1 (17)	C24—C23—C22	118.4 (2)
C10—C11—C12	120.95 (19)	C24—C23—H23	121.4 (16)
C10—C11—H11	122.8 (16)	C23—C24—H24	120.2 (17)
C12—C11—H11	116.2 (16)	C25—C24—C23	119.2 (2)
C11—C12—H12	122.3 (16)	C25—C24—H24	120.7 (17)
C13—C12—C11	118.25 (19)	C24—C25—H25	118.6 (17)
C13—C12—H12	119.5 (16)	C26—C25—C24	118.5 (2)
C8—C13—C14	106.09 (16)	C26—C25—H25	122.8 (17)
C12—C13—C8	120.70 (19)	N2—C26—C25	123.7 (2)
C12—C13—C14	132.98 (18)	N2—C26—H26	115.5 (16)
O1—C14—O2	109.19 (15)	C25—C26—H26	120.8 (16)
O1—C14—C13	103.65 (15)		
			
O1—C7—C8—C9	161.0 (2)	C8—C7—C22—N2	−61.9 (2)
O1—C7—C8—C13	−24.38 (19)	C8—C7—C22—C23	116.4 (2)
O1—C7—C22—N2	−176.09 (17)	C8—C9—C10—C11	−1.1 (3)
O1—C7—C22—C23	2.2 (3)	C8—C13—C14—O1	24.79 (19)
O1—C14—C15—N1	128.66 (18)	C8—C13—C14—O2	−91.61 (18)
O1—C14—C15—C16	−52.5 (3)	C8—C13—C14—C15	147.19 (17)
O2—C1—C2—C3	179.6 (2)	C9—C8—C13—C12	0.5 (3)
O2—C1—C6—C5	−179.70 (18)	C9—C8—C13—C14	175.61 (18)
O2—C1—C6—C7	−3.1 (3)	C9—C10—C11—C12	0.5 (3)
O2—C14—C15—N1	−113.89 (19)	C10—C11—C12—C13	0.6 (3)
O2—C14—C15—C16	65.0 (2)	C11—C12—C13—C8	−1.1 (3)
O3—N1—C15—C14	178.80 (15)	C11—C12—C13—C14	−174.7 (2)
O3—N1—C15—C16	−0.3 (2)	C12—C13—C14—O1	−161.0 (2)
N1—O3—C17—C16	−1.0 (2)	C12—C13—C14—O2	82.6 (3)
N1—O3—C17—C18	178.76 (19)	C12—C13—C14—C15	−38.6 (3)
N1—C15—C16—C17	−0.3 (2)	C13—C8—C9—C10	0.6 (3)
N1—C15—C16—C19	−177.3 (2)	C13—C14—C15—N1	9.4 (3)
N2—C22—C23—C24	−0.7 (3)	C13—C14—C15—C16	−171.76 (19)
C1—O2—C14—O1	−41.1 (2)	C14—O1—C7—C6	−70.50 (17)
C1—O2—C14—C13	71.8 (2)	C14—O1—C7—C8	40.23 (17)
C1—O2—C14—C15	−158.98 (16)	C14—O1—C7—C22	163.34 (15)
C1—C2—C3—C4	0.3 (4)	C14—O2—C1—C2	−174.96 (18)
C1—C6—C7—O1	35.3 (2)	C14—O2—C1—C6	4.8 (3)
C1—C6—C7—C8	−72.2 (2)	C14—C15—C16—C17	−179.22 (19)
C1—C6—C7—C22	156.07 (18)	C14—C15—C16—C19	3.8 (4)
C2—C1—C6—C5	0.0 (3)	C15—C16—C17—O3	0.8 (2)
C2—C1—C6—C7	176.65 (19)	C15—C16—C17—C18	−178.9 (3)
C2—C3—C4—C5	−0.3 (4)	C15—C16—C19—O4	171.3 (2)
C3—C4—C5—C6	0.1 (3)	C15—C16—C19—O5	−8.8 (3)
C4—C5—C6—C1	0.0 (3)	C17—O3—N1—C15	0.8 (2)
C4—C5—C6—C7	−176.3 (2)	C17—C16—C19—O4	−5.2 (3)
C5—C6—C7—O1	−148.31 (19)	C17—C16—C19—O5	174.7 (2)
C5—C6—C7—C8	104.1 (2)	C19—O5—C20—C21	−176.4 (2)
C5—C6—C7—C22	−27.6 (3)	C19—C16—C17—O3	178.15 (18)
C6—C1—C2—C3	−0.2 (3)	C19—C16—C17—C18	−1.6 (4)
C6—C7—C8—C9	−89.3 (2)	C20—O5—C19—O4	−8.2 (3)
C6—C7—C8—C13	85.24 (18)	C20—O5—C19—C16	171.90 (18)
C6—C7—C22—N2	65.2 (2)	C22—N2—C26—C25	−0.3 (4)
C6—C7—C22—C23	−116.5 (2)	C22—C7—C8—C9	43.0 (3)
C7—O1—C14—O2	76.13 (17)	C22—C7—C8—C13	−142.41 (17)
C7—O1—C14—C13	−40.49 (17)	C22—C23—C24—C25	−0.4 (4)
C7—O1—C14—C15	−168.89 (15)	C23—C24—C25—C26	1.1 (4)
C7—C8—C9—C10	174.6 (2)	C24—C25—C26—N2	−0.8 (4)
C7—C8—C13—C12	−174.74 (17)	C26—N2—C22—C7	179.4 (2)
C7—C8—C13—C14	0.4 (2)	C26—N2—C22—C23	1.1 (3)
C7—C22—C23—C24	−178.9 (2)		

### Hydrogen-bond geometry (Å, º)

**Table:**

*D*—H···*A*	*D*—H	H···*A*	*D*···*A*	*D*—H···*A*
C5—H5···N2	0.96 (2)	2.42 (2)	3.085 (3)	125.8 (18)
C23—H23···N1^i^	1.00 (3)	2.70 (3)	3.669 (3)	163 (2)
C12—H12···O1^ii^	0.94 (3)	2.56 (3)	3.469 (2)	162 (2)
C18—H18*A*···O4^ii^	0.94 (4)	2.71 (4)	3.297 (3)	121 (3)
C18—H18*B*···O4	0.99 (4)	2.38 (4)	3.040 (3)	123 (3)

Symmetry codes: (i) *x*, −*y*+1/2, *z*−1/2; (ii) *x*, −*y*+1/2, *z*+1/2.

## References

[bb1] Ando, Y., Hanaki, A., Sasaki, R., Ohmori, K. & Suzuki, K. (2017). *Angew. Chem. Int. Ed.* **56**, 11460–11465. 10.1002/anie.201705562 28671750

[bb2] Ando, Y., Tanaka, D., Sasaki, R., Ohmori, K. & Suzuki, K. (2019). *Angew. Chem. Int. Ed.* **58**, 12507–12513. 10.1002/anie.201906762 31278822

[bb3] Bruker (2016). *SAINT* and *APEX3*. Bruker AXS Inc., Madison, Wisconsin, USA.

[bb4] Dolomanov, O. V., Bourhis, L. J., Gildea, R. J., Howard, J. A. K. & Puschmann, H. (2009). *J. Appl. Cryst.* **42**, 339–341.

[bb5] Duncan, N. S., Beall, H. D., Kearns, A. K., Li, C. & Natale, N. R. (2014). *Acta Cryst.* E**70**, o315–o316. 10.1107/S1600536814003080 PMC3998422 24765016

[bb6] Filatov, M. A., Karuthedath, S., Polestshuk, P. M., Savoie, H., Flanagan, K. J., Sy, C., Sitte, E., Telitchko, M., Laquai, F., Boyle, R. W. & Senge, M. O. (2017). *J. Am. Chem. Soc.* **139**, 6282–6285. 10.1021/jacs.7b00551 28407710

[bb7] Groom, C. R., Bruno, I. J., Lightfoot, M. P. & Ward, S. C. (2016). *Acta Cryst.* B**72**, 171–179. 10.1107/S2052520616003954 PMC4822653 27048719

[bb8] Han, X., Li, C., Mosher, M. D., Rider, K. C., Zhou, P., Crawford, R. L., Fusco, W., Paszczynski, A. & Natale, N. R. (2009). *Bioorg. Med. Chem.* **17**, 1671–1680. 10.1016/j.bmc.2008.12.056 PMC2978248 19167892

[bb9] Han, X., Li, C., Rider, K. C., Blumenfeld, A., Twamley, B. & Natale, N. R. (2002). *Tetrahedron Lett.* **43**, 7673–7677.

[bb10] Han, X., Twamley, B. & Natale, N. R. (2003). *J. Heterocycl. Chem.* **40**, 539–545.

[bb11] Klaper, M., Wessig, P. & Linker, T. (2016). *Chem. Commun.* **52**, 1210–1213. 10.1039/c5cc08606j 26608846

[bb12] Krause, L., Herbst-Irmer, R., Sheldrick, G. M. & Stalke, D. (2015). *J. Appl. Cryst.* **48**, 3–10. 10.1107/S1600576714022985 PMC4453166 26089746

[bb13] Lauer, A., Dobryakov, A. L., Kovalenko, S. A., Fidder, H. & Heyne, K. (2011). *Phys. Chem. Chem. Phys.* **13**, 8723–8732. 10.1039/c0cp02218g 21331386

[bb14] Li, C., Campbell, M. J., Weaver, M. J., Duncan, N. S., Hunting, J. L. & Natale, N. R. (2013). *Acta Cryst.* E**69**, o1804–o1805. 10.1107/S1600536813031395 PMC4004437 24860293

[bb15] Li, C., Twamley, B. & Natale, N. R. (2006). *Acta Cryst.* E**62**, o854–o856.

[bb16] Li, C., Twamley, B. & Natale, N. R. (2008). *J. Heterocycl. Chem.* **45**, 259–264.

[bb17] McKinnon, J. J., Jayatilaka, D. & Spackman, M. A. (2007). *Chem. Commun.* pp. 3814–3816. 10.1039/b704980c 18217656

[bb18] Mosher, M. D., Natale, N. R. & Vij, A. (1996). *Acta Cryst.* C**52**, 2513–2515.

[bb19] Sheldrick, G. M. (2008). *Acta Cryst.* A**64**, 112–122. 10.1107/S0108767307043930 18156677

[bb20] Sheldrick, G. M. (2015). *Acta Cryst.* C**71**, 3–8.

[bb21] Spackman, M. A. & Jayatilaka, D. (2009). *CrystEngComm* **11**, 19–32.

[bb27] Turner, M. J., McKinnon, J. J., Wolff, S. K., Grimwood, D. J., Spackman, P. R., Jayatilaka, D. & Spackman, M. A. (2017). *CrystalExplorer17*. University of Western Australia. http://hirshfeldsurface.net

[bb22] Vander Heiden, M. G., Cantley, L. C. & Thompson, C. B. (2009). *Science*, **324**, 1029–1033. 10.1126/science.1160809 PMC2849637 19460998

[bb23] Walker, M., Pohl, E., Herbst-Irmer, R., Gerlitz, M., Rohr, J. & Sheldrick, G. M. (1999). *Acta Cryst.* B**55**, 607–616. 10.1107/s0108768199003948 10927402

[bb24] Weaver, M. J., Kearns, A. K., Stump, S., Li, C., Gajewski, M. P., Rider, K. C., Backos, D. S., Reigan, P. R., Beall, H. D. & Natale, N. R. (2015). *Bioorg. Med. Chem. Lett.* **25**, 1765–1770. 10.1016/j.bmcl.2015.02.063 PMC4599781 25782743

[bb25] Weaver, M. J., Stump, S., Campbell, M. J., Backos, D. S., Li, C., Reigan, P., Adams, E., Beall, H. D. & Natale, N. R. (2020). *Bioorg. Med. Chem.* **28**, 115781. 10.1016/j.bmc.2020.115781 PMC7933047 33038788

[bb26] Westrip, S. P. (2010). *J. Appl. Cryst.* **43**, 920–925.

