# Supplementary figures and images for: Ethyl 5-methyl-3-[11-(pyridin-2-yl)-6,11-di­hydro-6,11-ep­oxy­dibenzo[b,e]oxepin-6-yl]isoxazole-4-carboxylate: a bicyclic acetal from the rearrangement of an anthracenyl isoxazole
Source: Acta Crystallogr E Crystallogr Commun. 2020 Nov 6;76(Pt 12):1818–22. doi: 10.1107/S2056989020014358 (PMC7784648; doi:10.1107/S2056989020014358)

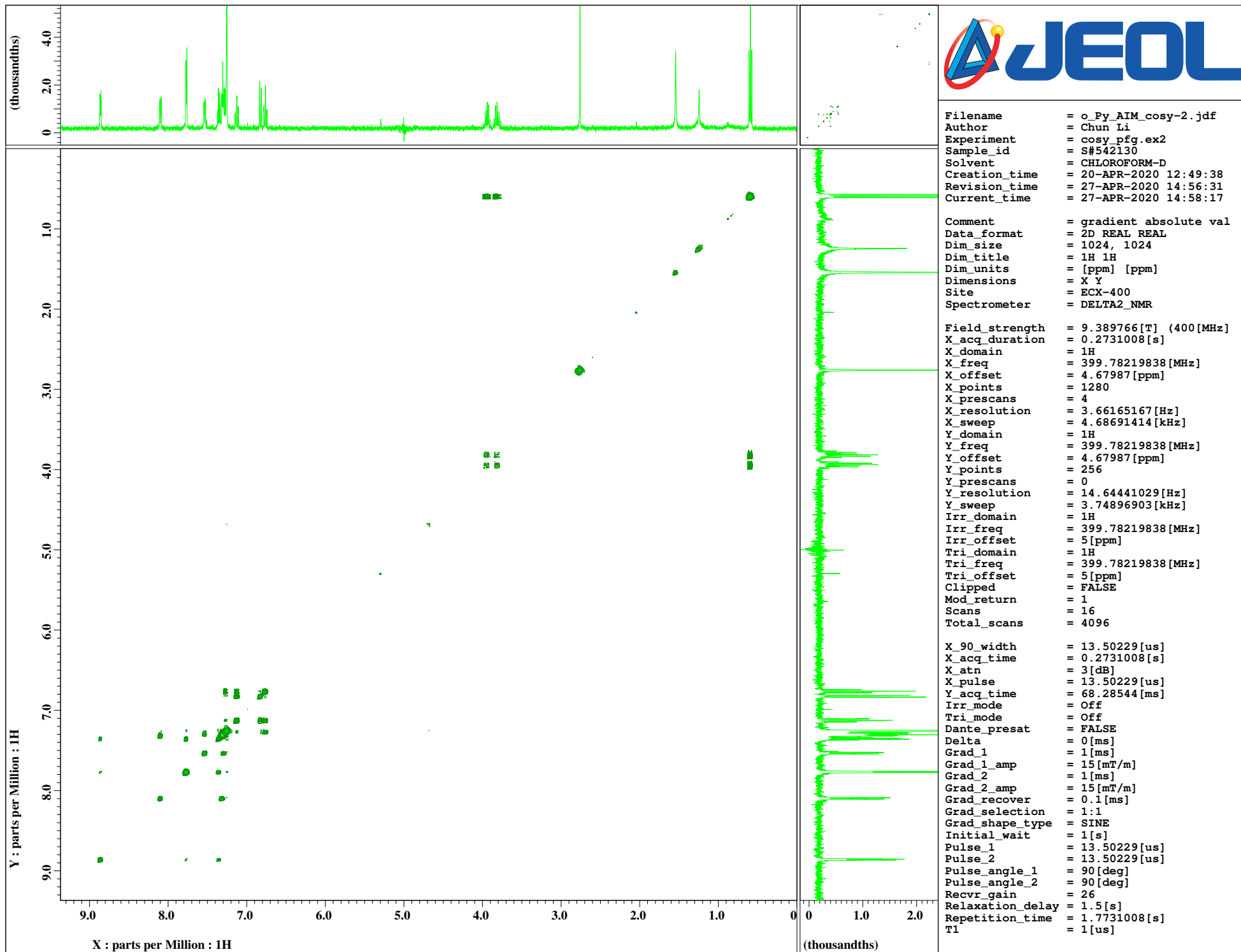

Supplement: Supplementary file 3 [file e-76-01818-sup3.pdf]
